# Mechanism of DNA loading by the DNA repair helicase XPD

**DOI:** 10.1093/nar/gkw102

**Published:** 2016-02-20

**Authors:** Diana Constantinescu-Aruxandei, Biljana Petrovic-Stojanovska, J. Carlos Penedo, Malcolm F. White, James H. Naismith

**Affiliations:** Biomedical Sciences Research Complex, University of St Andrews, Fife KY16 9ST, UK

## Abstract

The xeroderma pigmentosum group D (XPD) helicase is a component of the transcription factor IIH complex in eukaryotes and plays an essential role in DNA repair in the nucleotide excision repair pathway. XPD is a 5′ to 3′ helicase with an essential iron–sulfur cluster. Structural and biochemical studies of the monomeric archaeal XPD homologues have aided a mechanistic understanding of this important class of helicase, but several important questions remain open. In particular, the mechanism for DNA loading, which is assumed to require large protein conformational change, is not fully understood. Here, DNA binding by the archaeal XPD helicase from *Thermoplasma acidophilum* has been investigated using a combination of crystallography, cross-linking, modified substrates and biochemical assays. The data are consistent with an initial tight binding of ssDNA to helicase domain 2, followed by transient opening of the interface between the Arch and 4FeS domains, allowing access to a second binding site on helicase domain 1 that directs DNA through the pore. A crystal structure of XPD from *Sulfolobus acidocaldiarius* that lacks helicase domain 2 has an otherwise unperturbed structure, emphasizing the stability of the interface between the Arch and 4FeS domains in XPD.

## INTRODUCTION

XPD (xeroderma pigmentosum group D) is a 5′-3′ superfamily 2 (SF2) helicase (1) that unwinds damaged DNA during the process of nucleotide excision repair (NER). In eukaryotes, XPD is one of the components of transcription factor IIH (TFIIH) along with nine other protein subunits (2–6). The major enzymatic function of XPD is to unwind the DNA double helix around lesions such as photoproducts to allow repair (7). XPD acts as a structural bridge between the core subunits and the Cdk-activating kinase (CAK) complex (6,8). While TFIIH is essential for both transcription initiation and NER, the adenosine triphosphate (ATP)-dependent helicase activity of XPD is only required for repair (5,9,10). In humans, XPD mutations result in three related diseases: xeroderma pigmentosum (XP), trichothiodystrophy and combined XP with Cockayne's syndrome (XP/CS) (11).

**Figure 1.**
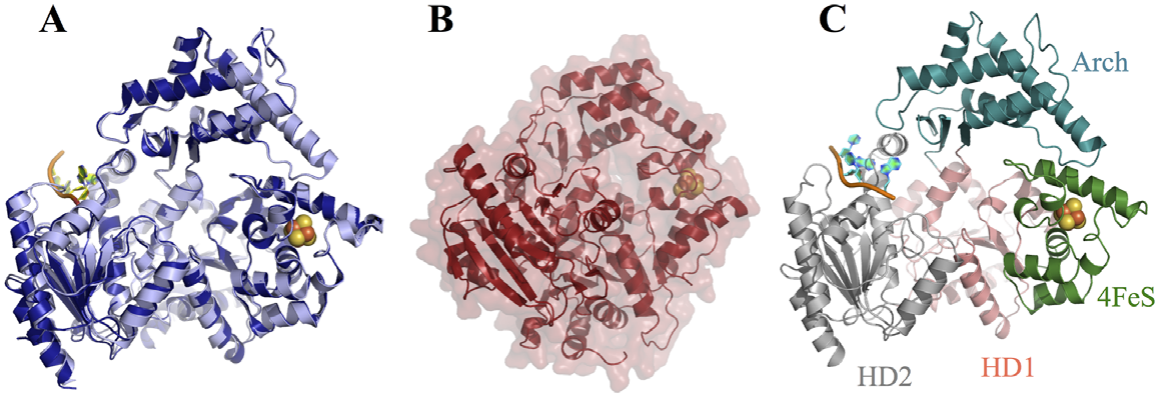
Crystal structures of XPD. (**A**) Cartoon representation of apo TaXPD (lightblue) (PDB ID: 2vsf) and TaXPD (density)—DNA (yellow) (PDB ID: 4a15). Both structures show the Arch domain in contact with the 4FeS domain resulting in closed conformation. (**B**) The surface representation of apo SaXPD shows that this homologue has practically no pore in the closed conformation. (**C**) Crystal structure of the covalent TaXPD–DNA complex showing each domain coloured differently: HD1—salmon, 4FeS—green, Arch—deepteal and HD2—grey. The 4Fe-4S cluster is shown as orange–yellow spheres in all structures.

The monomeric archaeal homologues of XPD have proven amenable to study, with four apo crystal structures (PDB IDs: 2vsf; 3crv; 3crw; and 2vl7) reported (12–14). Archaeal XPD is comprised of four domains: two RecA-like domains that form the motor core (HD1 and HD2) and two auxiliary domains (4FeS domain and Arch domain) that are inserted into HD1. The 4FeS domain is stabilized by a 4Fe-4S cluster that is essential for the helicase activity (15) and is conserved in a family of eukaryotic SF2B helicases (15). This cluster was suggested to have a role in charge transfer (CT) via DNA, which could be involved in lesion recognition and conformational control (16–18). The Arch, 4FeS and HD1 domains form a central ‘pore’ that is largely closed in the crystal structures (Figure 1A) and the presumed helicase mechanism of XPD involves the passage of the translocated strand through this ‘pore’ (12–14,19,20). However, in the case of *Sulfolobus acidocaldarius* XPD (SaXPD) the ‘pore’ does not actually exist when the molecule is viewed as a surface, rather a pore appears only in cartoon representations (Figure 1B). The *in vivo* function of XPD requires binding to nucleotides within a repair bubble, thus XPD would have to undergo a conformational change separating the Arch and 4FeS domains to create a pore as these domains are tightly packed in SaXPD (Figure 1B) and *Thermoplasma acidophilum* XPD (TaXPD) (a small pore) (Figure 1A).

To date, there is only one reported crystal structure of a XPD–DNA complex (PDB ID: 4a15) (19) and although an oligonucleotide 22-nt long was used for crystallization, only 4 nt were located, bound in a cleft in HD2. Mutational analysis suggested that the binding site of the translocated strand extended between the HD1 and 4FeS domains (19).

The mechanism of XPD helicase activity remains unclear with uncertainties about the binding of the 3′-end of the translocated DNA strand, the positioning of the junction between single- and double-stranded DNA and the role of protein conformational change in unwinding the DNA. In a recent study, the opening of the pore was monitored by attaching a Cy3 fluorophore to a cysteine mutant in the Arch domain of *Ferroplasma acidarmanus* XPD (FaXPD) and measuring the quenching by the 4Fe-4S cluster in a single molecule system (21). FaXPD was found to undergo transitions between the closed state and what was proposed to be an open state, both in the presence and absence of DNA. DNA was not observed to have any effect on the position of the equilibrium and the lifetime of the closed conformation was 3-fold longer than that of the open one. The apparent stability of the closed structure is consistent with the crystal structures, which are all closed—Figure 1A and B. The majority (70%) of DNA binding events were initiated in the closed conformation, suggesting that initial binding is not dependent on pore opening.

Here, we present a new TaXPD–DNA complex, obtained by covalently linking the 5′-end of the DNA to the protein through an alkanethiol moiety. We have experimentally identified further interactions between DNA and conserved residues of the protein. We demonstrate that DNA loading by TaXPD does not involve threading of DNA and that covalently linking the Arch domain to the 4FeS domain inhibits helicase activities without affecting the affinity of TaXPD for DNA. We propose a model for TaXPD loading onto and unwinding of DNA.

## MATERIALS AND METHODS

### TaXPD expression, purification and site directed mutagenesis

A synthetic gene of TaXPD, in which the three native cysteines that are not ligated to the iron cluster were mutated to alanine, was designed and purchased (DNA2.0, USA). The gene was supplied in the pJexpress401 vector with kanamycin resistance and a Tobacco Etch Virus (TEV)-cleavable N-terminal 6-histidine tag for affinity purification was included. This ‘no cysteine’ gene, in essence a new ‘native’, and subsequent mutants were transformed in *Escherichia coli* Rosetta cells (the sequences of mutagenic oligos are available from the corresponding author on request). For the crystallization experiments, a gene coding for a shorter construct (20–615), previously used for the apo structure (14) and that is missing the Q-motif, was more successful in obtaining good diffracting crystals. The missing Q-motif results in an enzyme that is less active as a helicase. However, since its ‘truncated’ constructs bind DNA with the same affinity as full length and recognizes ssDNA in the same manner as in the full length version study (19), we conclude it is a useful guide. All biological assays employed full-length protein.

The cells were grown in LB medium supplemented with 35 μg/ml kanamycin at 37°C. When OD_600nm_ reached 0.8–1, the temperature was lowered to 28°C and the protein expression was induced with 0.25 mM IPTG overnight. The cells were harvested (15 000 × *g*, 15 min, 4°C), resuspended and lysed by sonication in ice-cooled lysis buffer (20 mM Tris-HCl pH 7.5, 500 mM NaCl, 10 mM imidazole and one ethylenediaminetetraacetic acid (EDTA)-free protease-inhibitor tablet), followed by centrifugation at 40 000 × *g* at 4°C for 40 min. After passage through a 0.45 μm filter, the supernatant was loaded on a Ni-column equilibrated with the lysis buffer and the column was washed with buffer A (20 mM Tris-HCl pH 7.5, 500 mM NaCl, 30 mM imidazole) until the absorption reached the baseline. The proteins were eluted with an imidazole gradient running from 30 to 500 mM, with TaXPD generally eluting at around 170 mM imidazole. The fractions containing the protein were identified by sodium dodecyl sulphate-polyacrylamide gel electrophoresis (SDS-PAGE), pooled and dialysed for 2 h in 20 mM Tris-HCl pH 7.5, 500 mM NaCl, 1 mM Dithiothreitol (DTT). The His-tag was cleaved overnight by adding 0.1× w/w TEV protease in fresh buffer. Next day, the protein was loaded again on a Ni-column equilibrated with the lysis buffer without the protease inhibitor tablet and washed with the same buffer until the absorption reached the baseline. The cleaved protein, which does not bind to the column, was collected in the flow-through. The protein was concentrated down to 4–5 ml and loaded on a gel filtration column HiLoad 26/60 Superdex 200 column (GE Healthcare) equilibrated with gel-filtration (GF) buffer (20 mM Tris-HCl pH 7.5 or pH 8.2 (the E542C mutant), 200 mM NaCl). The protein-containing fractions were verified for purity by SDS-PAGE and the pure fractions were pooled and concentrated.

### Formation of the apo TaXPD–DNA crosslinked complex

Approximately 20–30 μM TaXPD E542C was incubated overnight at 4°C with a 2-fold excess of an alkanethiol 5′-modified DNA (IDT) in 20 mM Tris-HCl pH 8.3–8.5 + 50 mM NaCl, in the absence or presence of 1 mM AMPPNP/MgCl_2_. The buffer was degassed and purged with nitrogen and the tube was sealed with parafilm in an anaerobic glovebox under nitrogen, to reduce the oxygen level. The DNA sequence was 5′-HO-(CH_2_)_6_-S-S-(CH_2_)_6_-TAC GAC GGC CAG TGC-3′ for the 15mer. Shorter DNA lengths (9, 10, 11 and 13 nt—obtained by removing nucleotides from the 3′-end of the sequence above) and also one hairpin DNA (5′-HO-(CH_2_)_6_-S-S-(CH_2_)_6_-TAC GAG AGA GAG AGA ACC GAG CAT TTG CTC G-3′) were also used. Next, the sample was subjected to anion exchange chromatography using a MonoQ 5/50 GL column (Amersham Biosciences) eluted with a NaCl gradient from 50 mM to 1 M concentration. The free protein eluted at around 250 mM NaCl, the TaXPD–DNA complex at around 400 mM NaCl (the 13mer DNA) and the free DNA eluted at around 700 mM NaCl. The fractions were analysed by non-reducing SDS-PAGE. The fractions that eluted in the main peak of the complex were pulled together and concentrated to A_280_ ∼8. The sample was further diluted 1:1 with 20 mM Tris-HCl pH 8 to reduce the NaCl concentration to ∼200 mM for crystallization. The integrity of the complex was checked by mass spectrometry.

### Crystallization, data collection and structure analysis of the TaXPD–13mer DNA complex

The crosslinked complex was crystallized in an anaerobic glovebox under nitrogen at room temperature (RT). Crystals were obtained with sitting-drop vapour diffusion in drops containing equal volumes (1 + 1 μl) of the protein–DNA covalent complex and a reservoir solution containing 0.2 M KCl, 0.1 M Mg-Acetate × 4H_2_O, 0.05 M Na-cacodylate × 3H_2_O pH 6.5, 10% PEG8k (condition C2 of Natrix screen, Hampton Research). Prior to data collection, the crystal was soaked in a cryoprotecting solution consisting of the mother liquor supplemented with 2% PEG8k and 30% glycerol, mounted in a loop and immediately flash cooled in liquid nitrogen. Diffraction data were collected at Diamond Light Source, UK, beamline I24 at a wavelength of 0.9686 Å and beamsize 50 × 50 μm. In total 600 diffraction images were collected with 0.2° oscillation and 0.2 s exposure/image. The images were indexed and integrated with iMosflm (22,23), and scaled and merged with AIMLESS (24) to a 2.2 Å resolution. Molecular replacement with the PDB ID: 4a15 without the DNA and the 4Fe-4S cluster was used to phase the crystal structure with Phaser in CCP4 (25). The crystal belongs to space group P6_5_ with unit cell dimensions *a* = *b* = 78.6 Å and *c* = 177.8 Å. Refmac5 in CCP4 (26,27) was used for refinement. The parameters and statistics are shown in Table 1.

**Table 1.** Crystallographic data collection and statistics of TaXPD-13mer and SaXPD

Parameter	TaXPD-13mer PDB ID: 5H8W	SaXPD (truncated) PDB ID: 5H8C
***Data collection***		
Space group	P6_5_	P2_1_2_1_2_1_
Wavelength (Å)	0.9686	0.9686
Cell dimensions		
a, b, c (Å)	78.6, 78.6, 177.8	64.80, 77.8, 99.5
α, β, γ (deg)	90, 90, 120	90, 90, 90
Resolution (Å)	37.22–2.20 (2.27–2.20)	29.91–2.29 (2.35–2.29)
Total reflections	124929	110072
Unique reflections	31168	23154
Completeness (%)	99 (99.7)	99.6 (99.8)
Rmerge	8.8 (67.9)	3.3 (69.3)
<I/σI>	10.1 (2.4)	18.2 (2.3)
Redundancy	4.0 (4.0)	4.8 (4.9)
***Refinement***		
Resolution (Å)	37.22–2.20	29.91–2.29
Rcryst/Rfree (%)	20.8/24.3	20.3/23.9
No. of atoms		
Protein/ions/DNA/FeS	4805/2/85/8	2750/0/0/8
Water	43	7
Residual B factors (Å^2^)		
Protein/DNA	43/53	80/-
Water/ions	33/35	64/-
Rmsd Bond lengths (Å)	0.009	0.010
Rmsd Bond angles (º)	1.47	1.68
Ramach'n favoured (%)	98	96.1

### Intra-molecular crosslinking of Arch to 4FeS domain

Cysteine pairs 100C-238C and 107C-238C were introduced into the TaXPD protein to enable chemical crosslinking between the Arch and 4FeS domains. Approximately 30 μM TaXPD 100C-238C or 107C-312C was first incubated for ∼20 min with a 3× molar excess of Tris-(2-carboxyethyl)phosphine (TCEP) in order to reduce the cysteines. The excess of TCEP was removed with a PD10 or a PG25 desalting column (depending on the volume) equilibrated with GF buffer (pH 7.5). The protein concentration in each fraction was checked measuring the absorption at 280 nm with a nanodrop. Next, the protein was incubated in an anaerobic glovebox with 3–4 sequential additions of 0.5 × BM(PEG)_3_ (Thermo Scientific, Supplementary Scheme S1) excess, each subjected to ∼1 h incubation. A total of 10 mM β-mercaptoethanol was added to inhibit unreacted crosslinker and these chemicals were removed by passage over a PD10/PG25 desalting column equilibrated with gel filtration buffer (pH 7.5). Crosslinking efficiency was evaluated using a combination of mass spectrometry, 5, 5′-dithiobis-2-nitrobenzoic acid (DTNB or Ellman's reagent) assay and SDS-PAGE. The DTNB assay measures free thiol and thus reports the unreacted thiol that remains after crosslinking. The ‘no cysteine’ native TaXPD was used as control and its readings subtracted from the cysteine mutants. Extended overnight incubation followed by another hour incubation with 0.5× excess crosslinker improved crosslinking for 100–238C, but not 107–312C.

### DNA binding assay

The affinity between DNA and TaXPD was measured by fluorescence anisotropy with a Cary Eclipse fluorescence spectrophotometer (Varian) using excitation at 490 nm and emission at 520 nm. The pathlength was 1 cm; the emission and excitation slit widths were each 10 nm. Before protein titration, 100 nM hairpin DNA labelled with fluorescein at 3′-end (5′-TAC GAC AGA GAA GAG ACG AGC ATT TTT GCT CGG AAG GA-3′-Fl) was equilibrated in 120 μl buffer (20 mM 2-(N-morpholino)ethanesulfonic acid (MES) pH 6.4, 1 mM DTT and 1 mM MgCl_2_). The binding was monitored in the presence and absence of 1 mM non-hydrozable ATP analogue, AMPPNP. The protein stocks contained the same concentration of DNA to avoid its dilution. Anisotropy and total fluorescence intensity were measured in parallel after ∼2 min. equilibration following each protein addition and the effects of dilution on protein concentration were corrected. ‘Magic angle’ conditions were used in order to minimize rotational effects on fluorescence intensity. Data were fitted with the 1:1 binding quadratic equation, which is derived for total concentrations of reactants:

\begin{equation*} \begin{array}{*{20}l} {A = A_{{\rm min}} + (A_{{\rm max}} - A_{{\rm min}} ) \times } \hfill \\ {\frac{{\left( {100 + x + K_{\rm d} + \sqrt {\left( {100 + x + K_{\rm d} } \right)^2 - 4 \times 100 \times x} } \right)}}{{2 \times 100}}} \hfill \\ \end{array} \end{equation*}

where, *A*—anisotropy; *A*_min_—minimum anisotropy (anisotropy of free DNA); *A*_max_—maximum anisotropy (anisotropy of the TaXPD–DNA complex); *x*—total protein concentration; *K*_d_— the dissociation constant.

### ATPase assay

Adenosine triphosphatase (ATPase) activity was assayed with BIOMOL green reagent (28,29). The assay was performed in 20 mM MES pH 6.4 with the final volume 50 μl and a ssDNA with the sequence 5′-CGT CGA GGA ATT CAA CCA CCG CTC TTC TCA ACT GCA GTC TAG ACT CGA GC-3′ was used. The DNA and TaXPD concentrations were 25 and 50 nM, respectively. ATP and MgCl_2_ were added first to the plate followed by the protein–DNA mixture. The BIOMOL green reagent was added at different time points and the absorbance at 620 nm was recorded with a Multiskan FC (Thermo Scientific) after 5 min. incubation. Each ATPase assay was performed in triplicate and each measurement was accompanied by a standard curve using the provided phosphate sample. The ATPase activity of the Walker A mutant (K35A) was also investigated as a control.

### Helicase assay

The helicase activity was investigated using a fluorescence-based helicase assay and an open fork DNA adapted from a previous study (19). The DNA had a dabcyl modification on the 3′-end of the translocated strand (5′- AGC TAC CAT GCC TGC ACG AAT TAA GCA ATT CGT AAT CAT GGT CAT AGC T-3′-dabcyl) and a Cy3 label at the 5′-end of the opposite strand (Cy3–5′-AGC TAT GAC CAT GAT TAC GAA TTG CTT GGA ATC CTG ACG AAC TGT AG-3′). The oligonucleotides were purchased from IDT. The underlined parts form the duplex. In the duplex, dabcyl resided in close vicinity to and was quenched by Cy3. This quenching is removed upon unwinding by TaXPD. The substrate having the 5′-end of the translocated strand modified with biotin ± neutravidin was also tested. The oligonucleotides were annealed at a molar ratio of 1:1 (final concentration 40 μM each) in annealing buffer (10 mM Tris-HCl pH 7.5, 50 mM NaCl) by slowly cooling the sample overnight after 5 min incubation at 95°C in a water bath. The DNA was next run on a native 12% acrylamide:TBE gel. The visible fluorescent band was excised and cut in small pieces and slowly shaken overnight at 4°C in annealing buffer. The DNA solution was separated from the gel pieces with a micro bio-spin column (Bio-rad) and the DNA concentration was measured from the absorbance at 260 nm using the extinction coefficient 150 000 l mole^−1^ cm^−1^. The final concentration of the DNA substrate in the assay was 50 nM. Excess concentrations of TaXPD over DNA were necessary for efficient helicase activity. Assays were carried out in a Cary Eclipse fluorescence spectrophotometer (Varian), at 20°C in 20 mM MES pH 6.4 and in the presence of 0.1 mg/ml bovine serum albumin, 1 mM MgCl_2_ and 1 mM ATP, with a final total volume of 150 μl. All components except ATP were mixed and incubated together at RT for 10 min; ATP (15 μl of 10 mM in MES buffer) was added and incubated in the cuvette for the same period of time and the reaction was started by adding the mixed components in the cuvette. As a control, the inactive TaXPD Walker A mutation (K35A) was measured. For 100C-238C, the dependence on the degree of crosslinking was also tested.

### Expression, purification and crystallization of *S. acidocaldarius* XPD (SaXPD)

The xpd gene from *S. acidocaldarius* (1–551) cloned into the pET28c vector (Rudolf, 2006) was cut with BamHI/NcoI restriction enzymes and recloned into previously designed pEHISTEV vector (30) with kanamycin resistance. The three native cysteines that do not coordinate the 4Fe-4S cluster—C360, C523 and C543 were mutated to serine. SaXPD was expressed as an N-terminally 6His-tagged protein in *E. coli* C43 cells. The cells were grown in LB medium supplemented with 35 μg/ml kanamycin at 37°C. When OD_600nm_ reached 0.8–1, the temperature was lowered to 28°C and the protein expression was induced with 0.2 mM IPTG overnight. The purification was similar to TaXPD, the GF buffer having pH 7.5.

For crystallization, the protein was mixed with 1.4-fold excess of a 16T oligo having a fluorescein attached to the T8 nucleotide in 20 mM Tris pH 7.5, 100 mM NaCl, 1 mM MgCl_2_, 1 mM TCEP. The mixture was incubated at RT in an anaerobic glovebox under nitrogen for 1 h. Crystals were obtained with sitting-drop vapour diffusion in drops containing equal volumes (1 + 1 μl) of the protein–DNA complex and a reservoir solution containing 0.1 M Hepes pH 7.5, 5% isopropanol and 10% PEG4k (condition 41 from Crystal Screen Lite, Hampton Research). The crystals took an unexpectedly long time of ∼3 months to grow. Prior to data collection, the crystal was soaked in a cryoprotecting solution consisting of the mother liquor supplemented with 5% isopropanol, 2% PEG4k and 25% glycerol, mounted in a loop and immediately flash cooled in liquid nitrogen. Diffraction data were collected at Diamond Light Source, UK, beamline I24 at a wavelength of 0.9686 Å. 900 images of 0.15° oscillations were indexed, integrated, scaled and merged to a 2.3 Å resolution with the automated xia2 data-processing suite (31–36) available at Diamond. Three ensembles consisting of HD1 (10–81, 149–192 and 304–353), 4FeS (82–148) and Arch domain (193–303), respectively, were used to phase the crystal structure with Phaser in CCP4 (25). The crystal belongs to space group P2_1_2_1_2_1_ with unit cell dimensions of *a* = 64.8 Å, *b* = 77.8 Å and *c* = 99.5 Å. Refmac5 in CCP4 (26,27) was used for refinement. The parameters and statistics are shown in Table 1.

## RESULTS

### TaXPD–DNA covalent complex

**Figure 2.**
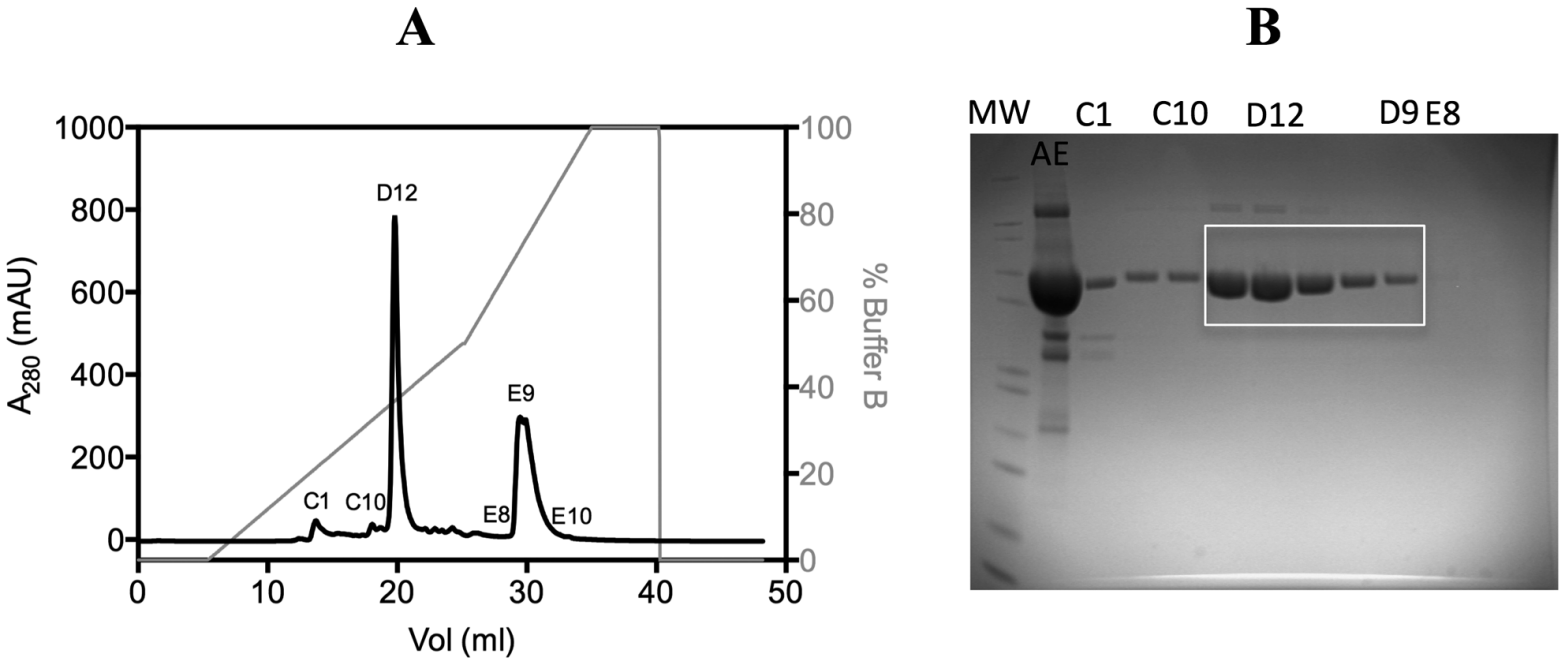
(**A**) Anion exchange (AE) purification of TaXPD E542C–13mer covalent complex. The peak corresponding to fraction C1 contains the apo TaXPD; the peak corresponding to D12 and the one between E8 and E10 are the elution of the covalent complex and apo DNA, respectively (see panel **B**). The grey line represents the gradient of buffer B. (B) SDS-PAGE with samples corresponding to the main peaks of the AE purification; MW is the molecular marker and AE corresponds to the sample loaded onto the MonoQ column. One can see the shift in mass between the apo protein and the complex (best seen between fractions C1 and C10).

Initial attempts to obtain crystals of an XPD–DNA complex, with protein from three archaeal species (*Sulfolobus tokodaii, S. acidocaldarius* and *Thermoplasma acidophilium*) and different DNA sequences with or without a fluorescein modification yielded the native protein structure. Therefore, we decided to covalently crosslink the DNA to the protein through disulphide trapping, inspired by previous work (37–41) although we used the alkanethiol moiety at the 5′ end of DNA. Based on the available structure of a TaXPD–DNA complex (19), we inserted a E542C mutation in the new ‘no cysteine’ native protein to form an intermolecular disulphide between TaXPD and alkanethiol-modified oligonucleotide DNA (Supplementary Figure S1). Our prediction was that the crosslinker would be flexible enough to allow the DNA to adopt the conformation seen previously at the 5′ end and by anchoring the DNA we would disclose the 3′ binding region. The crosslinking reaction was efficient, with more than 80% of protein-modified covalent complex (Figure 2 and Supplementary Figure S2). Similar results were obtained for ssDNA of length varying from 9 (Supplementary Figure S3A) to 15 nt as well as a hairpin DNA (Supplementary Figure S4). This crosslinking method has the advantage that the oligonucleotides can be synthesized commercially.

Good diffracting crystals of all complexes tested were obtained only in the presence of Mg^2+^ and after longer periods of time (weeks) compared with the protein in the absence of covalently linked DNA or in the presence of unmodified DNA (days). All crystal structures showed electron density truncated around 4–5 nt. Mass spectrometry revealed upon overnight incubation with MgCl_2_ (required for crystallization) that DNA was cleaved to shorter forms (Supplementary Figure S3B). Higher MgCl_2_ concentrations (10 mM) resulted in a TaXPD-7mer when AMPPNP was present and a TaXPD-5mer when it was not (Supplementary Figure S3C). The TaXPD–DNA complex was preserved in the presence of AMPPNP and MgCl_2_ when using a 3′-hairpin DNA (Supplementary Figure S4C). We attribute the cleavage to very low levels of a contaminating nuclease activity and to the 3′ end of the DNA being mobile.

**Figure 3.**
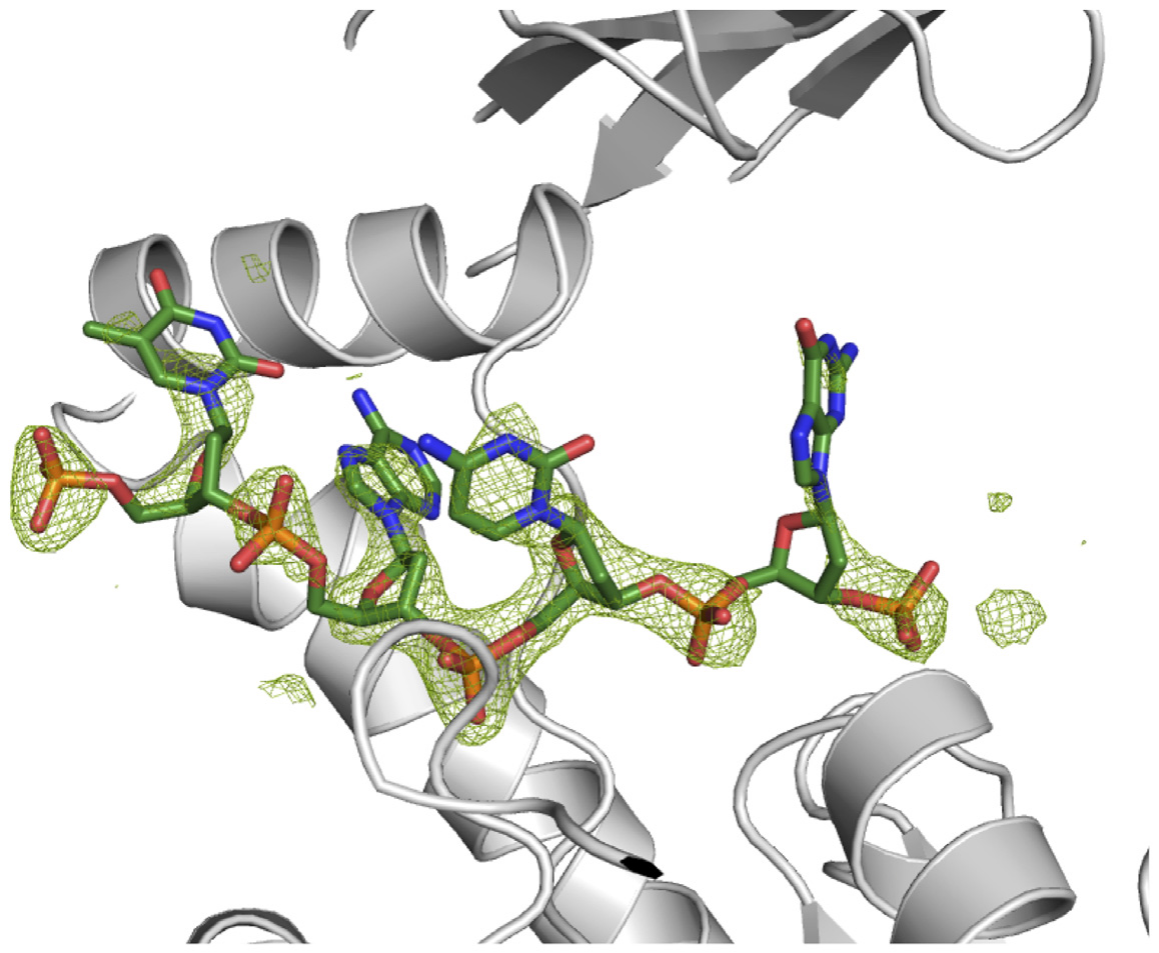
Fo-Fc map of DNA contoured at 3 σ after refinement of the protein structure; the DNA structure is modelled into the electronic density.

**Figure 4.**
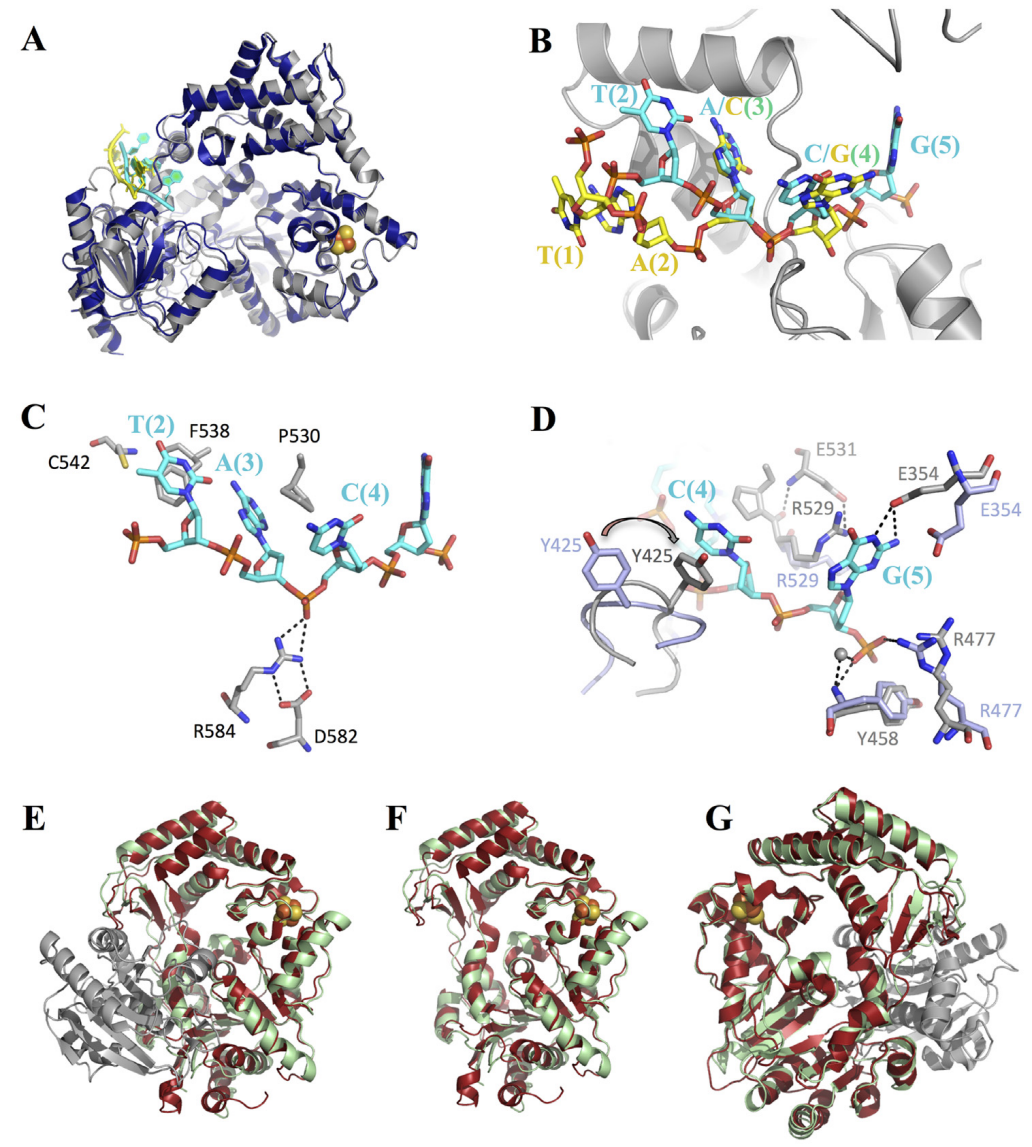
(**A**) Covalent TaXPD–DNA (grey: TaXPD, cyan: DNA) in comparison with previous TaXPD–DNA complex structure, PDB ID: 4a15 (density: TaXPD, yellow: DNA). (**B**) Closeup view of the covalently crosslinked DNA (cyan) to TaXPD (grey) in comparison with the previous DNA conformation (yellow). The positions of nucleotides relative to TaXPD corresponding to PDB ID: 4a15 are shown in brackets. The nucleotide in position 2 (T) is shifted upwards compared with the corresponding nucleotide (A) in PDB ID: 4a15. (**C**) Preserved interactions in the covalent complex (D582–R584–phosphate polar contacts and DNA base (A) stacked between F538 and P530); same colour code as in (B). (**D**) New structural information derived from the covalent complex, not reported previously: the loop 422–428 changes its conformation compared with the apo structure (PDB ID: 2vsf) as Y425 interacts with the cytosine base; the guanine base is stabilized by R529 and E354 and the phosphate through hydrogen bonds with the NH- of Y458, a water molecule and possibly R477; lightblue: the apo TaXPD; grey and cyan: TaXPD and DNA in the covalent complex, respectively. (**E**) Crystal structure of SaXPD with the HD2 domain proteolytically cleaved (palegreen) in comparison to full-length SaXPD, PDB ID: 3crv (red: HD1, 4FeS, Arch and grey: HD2); (**F**) Front side of SaXPD, HD2 (the cleaved domain) is not shown for clarity; (**G**) ∼180° rotation of Figure 4E showing comparison of SaXPD full-length and the cleaved protein.

The highest quality structure was obtained from a crystal of the TaXPD–13mer covalent complex diffracting to 2.2 Å resolution. Unambiguous unbiased electron density was observed for the 4 nt (TACG) from the 5′- end of the DNA after refinement of the protein (Figure 3). The density improved after modelling each nucleotide into the density. Additionally, some density for the—(CH)_6_—linker and weak density of the fifth nucleotide (adenosine) that cannot be clearly interpreted appeared after refinement of the first four nucleotides (Supplementary Figure S5). The overall conformation of TaXPD is similar to the previous structure of a TaXPD–DNA complex, PDB ID: 4a15 (19), with the Arch domain in contact to the 4FeS domain (Figures 1C and 4A). This suggests that the crosslink does not disrupt the canonical DNA binding interface. Three nucleotides approximately overlap with the previous structure (Figure 4A), but the N position corresponds to the N + 1 position from the 5′ end in the previous structure (19). Therefore, our fourth nucleotide (guanine) occupies the fifth position not seen before (Figure 4B), disclosing new interactions. For clarity, the nucleotides’ positions in the PDB ID: 4a15 instead of the numbers will be used henceforth.

The nucleotide in position 2 from the 5′-end is shifted in our structure relative to the previous structure (19) presumably as a result of the crosslink (Figure 4B). Interactions reported previously (19) are preserved in our structure (Figure 4C): the Nη1 and Nη2 atoms of R584 stabilize the phosphate of cytosine in position 4 (previously guanine), D582 forms hydrogen bonds with R584 stabilizing it in the proper position for DNA binding, and the adenine base in position 3 (previously cytosine) is stacked between F538 and P530.

The Y425 residue in the loop 422–428 that could not be resolved in the previous complex interacts with the cytosine base from position 4. The loop undergoes a conformational change relative to the apo protein, which positions Y425 in a suitable conformation to stack against the cytosine base (Figure 4D). The nucleotide (G) corresponding to the fifth unresolved position in the previous complex structure is stabilized by the side chain of R529 stacking against the guanine base that also forms hydrogen bonds with E354 (Figure 4D). The phosphate in the sixth position is stabilized by polar interactions with the—NH of Y458 through a water molecule and probably by R477. Y458 is strictly conserved among several organisms (Y542 in human XPD) and Y542C is one of the mutants that cause the XP disease in humans.

### Crystal structure of a proteolytic fragment of SaXPD suggests rigidity in the HD1-Arch-4FeS substructure

The crystal structure of SaXPD, obtained from crystals that grew after an unexpectedly long (3 months) time, revealed the absence of the HD2 domain, which most probably was proteolytically cleaved. Interestingly, the rest of the domains maintained the closed conformation (Figure 4E–G), with the Arch domain being only slightly shifted compared to the full-length protein, probably due to lost interactions between the Arch domain and HD2 domain. The crystal packing positioned the Arch domain from a symmetric molecule in place of the missing HD2 domain.

### The role of the pore

The DNA–protein crystallization experiments suggested to us that ssDNA engages HD2 but is weakly (if at all) recognized by HD1 and this allows its facile degradation by contaminating nuclease(s). The current accepted model for DNA unwinding has DNA in contact with HD1. To experimentally establish the route of DNA, the Arch domain was covalently crosslinked to the 4FeS domain with BM(PEG)_3_ (1, 8-bis (maleimido) diethylene glycol, Scheme S1) to form a permanently closed enzyme (Supplementary Figure S6A and B). Two double cysteine mutants were designed and crosslinked: E107C (4FeS domain)—E312C (Arch domain) and D100C (4FeS domain)—R238C (Arch domain) with the distances between the Cβ of the cysteines 12 and 12.9 Å, respectively (Supplementary Figure S6C and D, respectively). Following crosslinking with BM(PEG)_3_, the percent of crosslinked cysteines was deduced from measurement of remaining free cysteines using a DTNB assay (Supplementary Figure S7A). A total of 17% of the cysteines of 107C-312C and 8% of 100C-238C remained unmodified (Supplementary Table S1) implying that the crosslinked protein was 83 and 92% of the total concentration, respectively. SDS-PAGE (Supplementary Figure S7B) shows that only a small amount of protein was crosslinked inter-molecularly and mass spectrometry confirmed monomeric TaXPD + one molecule of crosslinker as the dominant species (Supplementary Figure S8).

**Figure 5.**
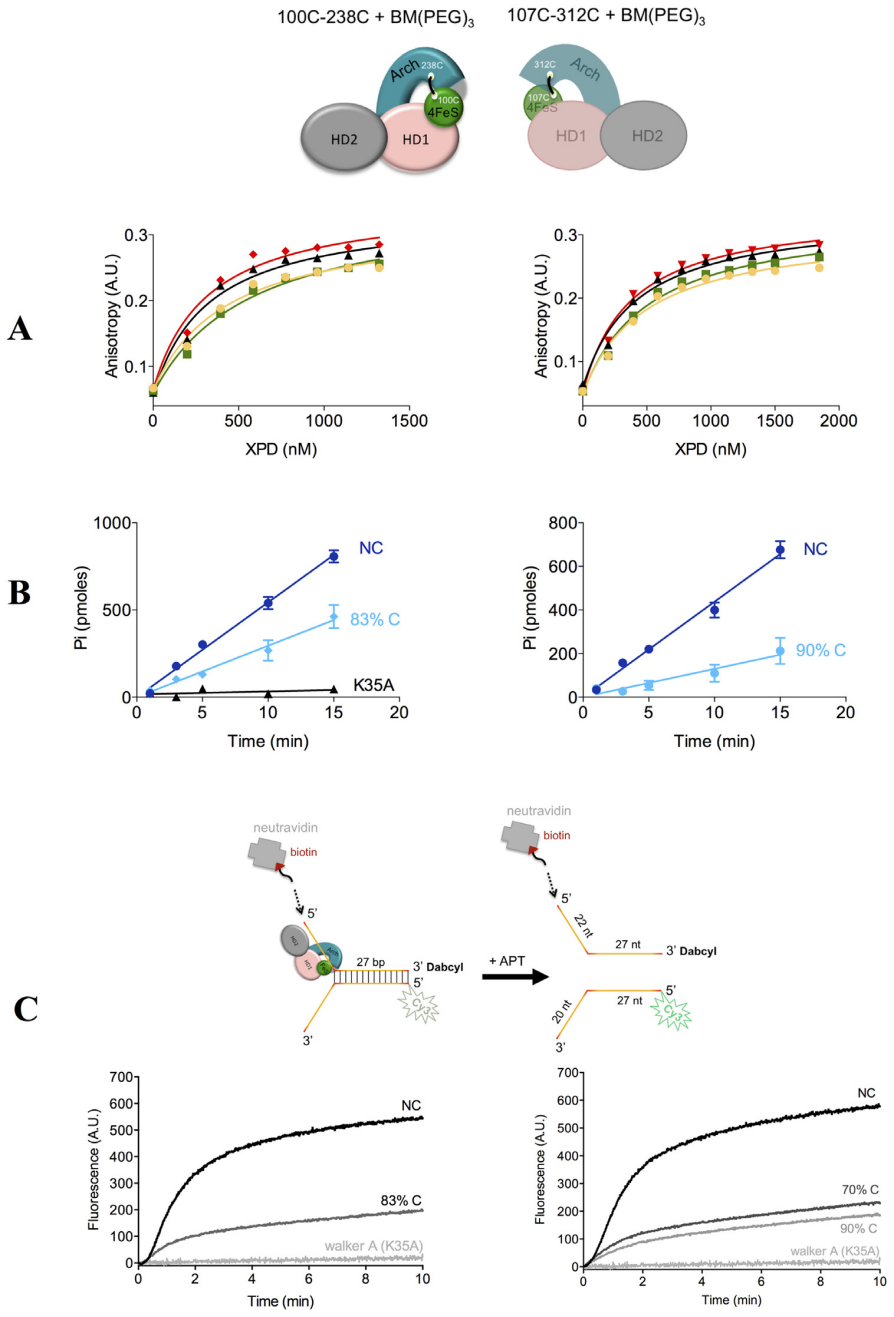
Biochemical data of intra-molecular crosslinked TaXPD (schematically represented above) in comparison to non-crosslinked TaXPD. (**A**) Fluorescence anisotropy data of DNA-binding in the absence (left) and presence (right) of 1 mM AMPPNP and the best-fit binding curves: 107–312 NC (black triangles), 107–312 83% C (red rhombs), 100–238 NC (yellow circles), 100–238 90% C (green squares). (**B**) ATPase assay of crosslinked and non-crosslinked TaXPD; left: 107–312 NC (blue), 107–312 83% C (cyan) and walker A mutant (K35A) (black); right: 100–238 NC (blue) and 100–238 90% C (cyan). (**C**) Time courses of DNA unwinding by intra-molecular crosslinked and non-crosslinked TaXPD 107C-312C (left) and 100C-238C (right). The walker A mutant (K35A) is also shown for comparison. A schematic representation of the substrate used in the helicase assays is shown above. The additional biotin-neutravidin modification on the 5′- overhang is modelled by arrows. NC: non-crosslinked, C: crosslinked. The percent of crosslinked TaXPD is also indicated.

These modified enzymes were assessed for DNA binding, ATPase and helicase activity (Figure 5A, B and C, respectively). The DNA binding affinities of the crosslinked species were not significantly different from the non-crosslinked controls (Figure 5A, Table 2), however DNA-stimulated ATPase activity of TaXPD was reduced by 2–3-fold (Figure 5B, Table 2). Helicase activity increased with the protein concentration up to 1 μM TaXPD (Supplementary Figure S9A). The time courses displayed clear lag phases, which suggests that TaXPD may proceed through multiple repeated steps to fully unwind the duplex DNA, as observed for other helicases (42). The intra-molecular crosslinked mutants, 100C-238C and 107C-312C, showed a large reduction (but not abolition) of the helicase activity (Figure 5C and Supplementary Figure S9A). The decrease in the helicase activity of 100C-238C appeared proportional with the degree of crosslinking (Figure 5C). Both biotin-modified DNA and neutravidin biotin-DNA (which is too big to pass through any conceivable pore) complex behaved normally in the helicase assay, ruling out DNA threading as a model (Supplementary Figure S9B).

**Table 2.** Effect of pore crosslinking on DNA binding and ATPase activity of TaXPD

Variant^a^	*K*_D1_ (μM)	*K*_D2_ (μM)	ATPase activity (mol ATP*min^−1^*mol^−1^ XPD)
100–238 NC	0.4 ± 0.1	0.5 ± 0.1	17.4 ± 0.5
100–238 C	0.6 ± 0.1	0.5 ± 0.1	5.2 ± 1.5
107–312 NC	0.3 ± 0.05	0.4 ± 0.05	21.1 ± 0.8
107–312 C	0.3 ± 0.1	0.4 ± 0.05	11.5 ± 1.9

^a^NC: non-crosslinked; C: crosslinked (91% for 100–238 and 83% for 107–312); *K*_D1_, *K*_D2_: dissociation constants of TaXPD–DNA in the absence and presence of AMPPNP, respectively.

## DISCUSSION

Studies of the archaeal XPD helicases have yielded a number of significant insights relevant to this important family of DNA repair enzymes. These have included the identification of the essential 4FeS cluster (15), structural and biochemical studies of archaeal XPDs that explained the molecular basis for disease causing mutations in humans (12–14,19), single-molecule studies of XPD mechanism (reviewed in (43)), the investigation of DNA damage detection (44–46) and the possibility of CT from the 4FeS cluster to DNA (reviewed in (47)). Nonetheless, the mechanism of XPD helicase, including such fundamental aspects as the precise path that the DNA adopts, is still not fully understood. It is presumed that the translocated strand passes through a central pore formed by the HD1, 4FeS and Arch domains. Yet all structures show that these domains make a number of polar and hydrophobic interactions that hold together the domains. Moreover, a spin labelling/PELDOR study of TaXPD yielded no evidence for opening at the interface between the Arch and 4FeS domain (48). Since XPD binds ssDNA within a repair bubble without a free DNA end, either the protein has to undergo a large conformational change separating the two domains or the DNA does not pass through the pore. Since no structure exists of a complex with a DNA fork, it is at least theoretically possible that the path of the translocated DNA beyond HD2 is non-canonical in this class of helicases.

Remarkably the structure of SaXPD with HD2 missing adopts the same closed state as the full-length protein, consistent with its stability, recently highlighted by spin labelling and mutational analysis of residues at this interface in TaXPD (48). Moreover, it was previously shown (12,13) that the conformation of the Arch domain was not affected by the 4FeS domain being disordered and thus by the disrupted interface between the two (PDB IDs: 3CRW and 2VL7). In contrast, the integrity of the FeS cluster/domain is greatly affected when not stabilized by the contact with the Arch domain (48). Overall, the data indicate a highly stable closed conformation of the pore.

Crystallizing protein–DNA complexes is often challenging due to low affinity or dynamic nature of the complexes. In some cases, this has been successfully overcome by covalently crosslinking internal sites of the DNA to the protein (37–41). Here we have presented a similar approach applied to the TaXPD helicase, but crosslinking the 5′-end instead of internal sites of DNA. The covalent TaXPD–DNA complex agrees with the previous reported TaXPD–DNA structure (19), in that it shows the strand bound to the HD2 domain. The Fo-Fc electron density permits the experimental positioning of an additional 3′ nucleotide compared to the previous study. We emphasize that the crosslinked protein was used only for structural studies not for functional ones. The data show degradation of the remaining bases in our system rather than disorder, whether this occurs in other systems is not known. Although a hairpin DNA was stable against nuclease cleavage we were unable to obtains crystals of the complex.

We observed a conformational change in loop 422–428 that allows Y425 to stack against the base in a π–π interaction not seen previously. Mutation of this conserved residue had been shown to result in decreased DNA affinity, helicase and ATPase activity (19). The corresponding loop in *S. acidocaldarius* and *S. tokodaii* XPD is already in the right conformation for DNA binding and the role of Y425 is probably accomplished by Y370 and R363, respectively, according to the structures. In human XPD, the equivalent of Y425 is F508, with probably similar structural function. R529 from HD2 and E354 from the Arch domain interact with the additional guanine base of DNA in our structure (Figure 4D). The corresponding residue of R529 in human XPD is Y527, which is probably also involved in stabilizing DNA bases.

**Figure 6.**
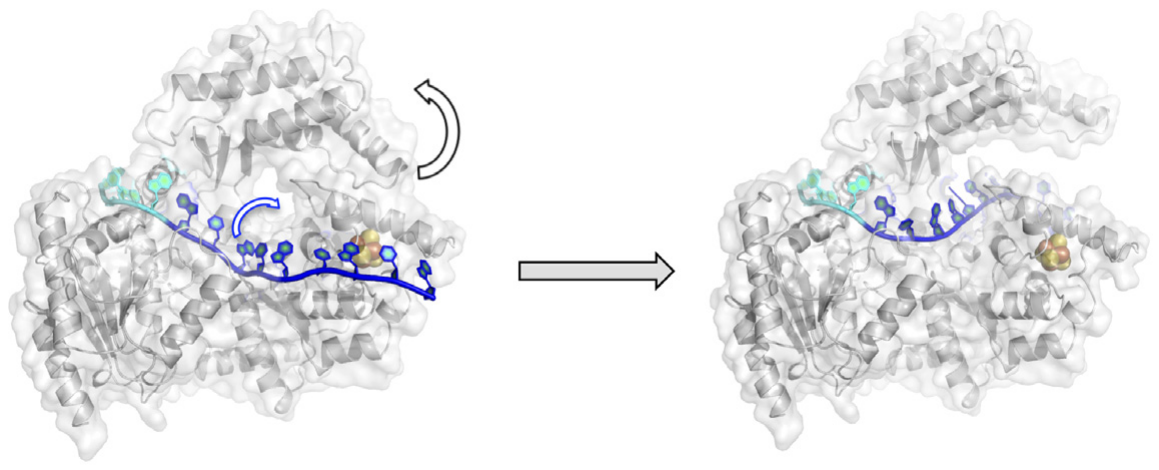
Representation of possible XPD–DNA conformational states. Initially, HD2 domain grabs the 5′-end of the DNA (shown in cyan), protecting it against nuclease degradation. The 3′-end of DNA is dynamic, prone to nuclease cleavage and might shuttle between in and out of the pore. The Arch–4FeS interface needs to get disrupted transiently for the DNA to pass through the pore during DNA translocation.

We show here for the first time that locking the Arch and 4FeS domains in the closed state through covalent crosslinking inactivates the TaXPD helicase activity (Figure 5C and Supplementary Figure S9A), although the DNA binding is unaffected (Figure 5A, Table 2) and the ATPase activity (Figure 5B, Table 2) is only reduced. We took care to control for residual helicase activity arising from non-crosslinked protein and made two different crosslinked variants. The preservation (albeit reduced) of ATPase activity shows that the crosslink has not grossly altered the protein structure. The conservation of DNA binding affinity is consistent with an essentially native structure for the crosslinked protein but also suggests that DNA makes few additional interactions (or only very weak ones) inside and at the other side of the pore region, correlating with the structural data. We used biotin-modified DNA analogues to rule out threading of DNA through the pore. The inactivity of the crosslinked proteins in the helicase assay is due to their inability to undergo the conformational change necessary to translocate DNA (DNA binding and ATP hydrolysis remain intact). These data represent strong experimental evidence that DNA is translocated through the pore, following an initial binding event that involves stable interactions of ssDNA with the HD2 domain, probably whilst the pore is conformationally closed (Figure 6), as proposed previously (21). Subsequently, transient opening of the interface between the Arch and 4FeS domains allows access to the pore and the secondary binding site along the top of the HD1 domain, as observed in other superfamily 2 helicases (49).

## ACCESSION NUMBERS

PDB IDs: 5H8C, 5H8W, 2vsf, 3crv, 3crw, 2vl7 and 4a15.

## Supplementary Material

SUPPLEMENTARY DATA
