# Supplementary material for: Mechanism of DNA loading by the DNA repair helicase XPD
Source: Nucleic Acids Res. 2016 Feb 20;44(6):2806–15. doi: 10.1093/nar/gkw102 (PMC4824113; doi:10.1093/nar/gkw102)
Supplement: SUPPLEMENTARY DATA [file supp_44_6_2806__index.html]

Mechanism of DNA loading by the DNA repair helicase XPD — SUPPLEMENTARY DATA 

# Mechanism of DNA loading by the DNA repair helicase XPD

## SUPPLEMENTARY DATA

- SUPPLEMENTARY DATA
