# Supplementary material for: Mechanism of DNA loading by the DNA repair helicase XPD
Source: Nucleic Acids Res. 2016 Feb 20;44(6):2806–15. doi: 10.1093/nar/gkw102 (PMC4824113; doi:10.1093/nar/gkw102)
Supplement: SUPPLEMENTARY DATA [file supp_gkw102_nar-03742-d-2015-File008.pdf]

Supporting material for:

Mechanism of DNA loading by the DNA Repair helicase XPD

Diana Constantinescu-Aruxandei, Biljana Petrovic-Stojanovska, Carlos Penedo-Esteiro, Malcolm F. White and James H. Naismith

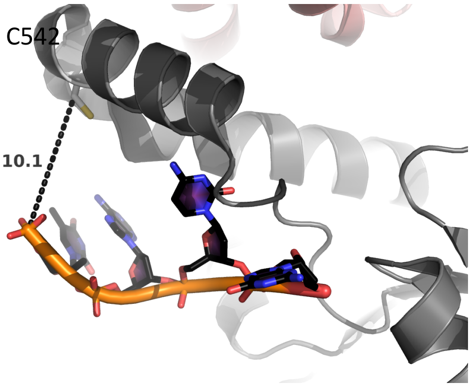

Figure S1. The distance between the cysteine mutant, E542C, used for the inter-molecular crosslinking of DNA to XPD and the 5'-end phosphate of DNA in the previous published complex (PDB 4a15).

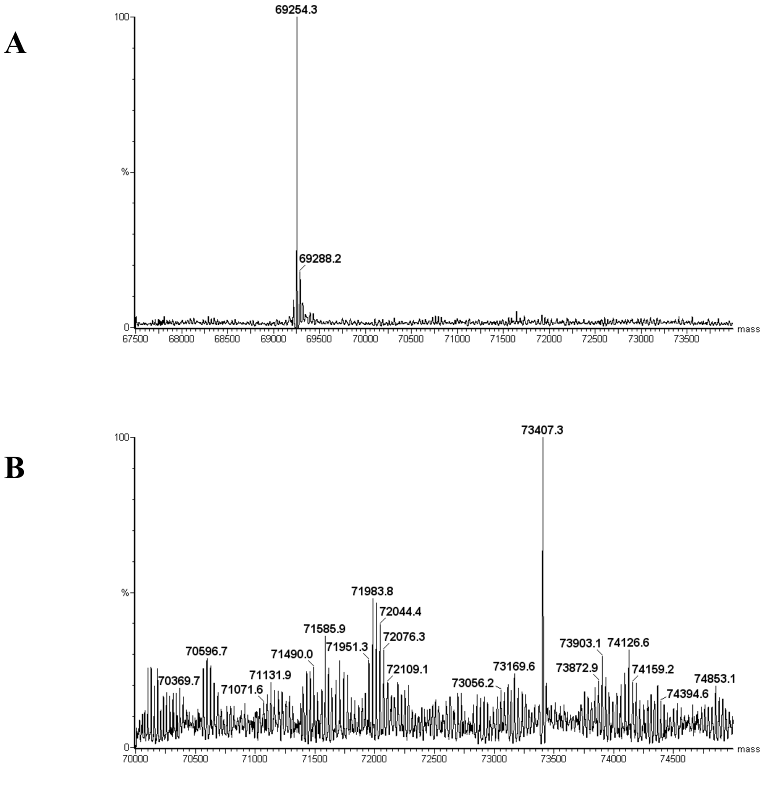

Figure S2. Intact mass spectrometry of apo XPD **(A)** and XPD-13mer covalent complex **(B)**. The theoretical molecular mass of the DNA + linker that should get attached to XPD is 4154 Da; the difference between the experimental masses is 4153 Da.

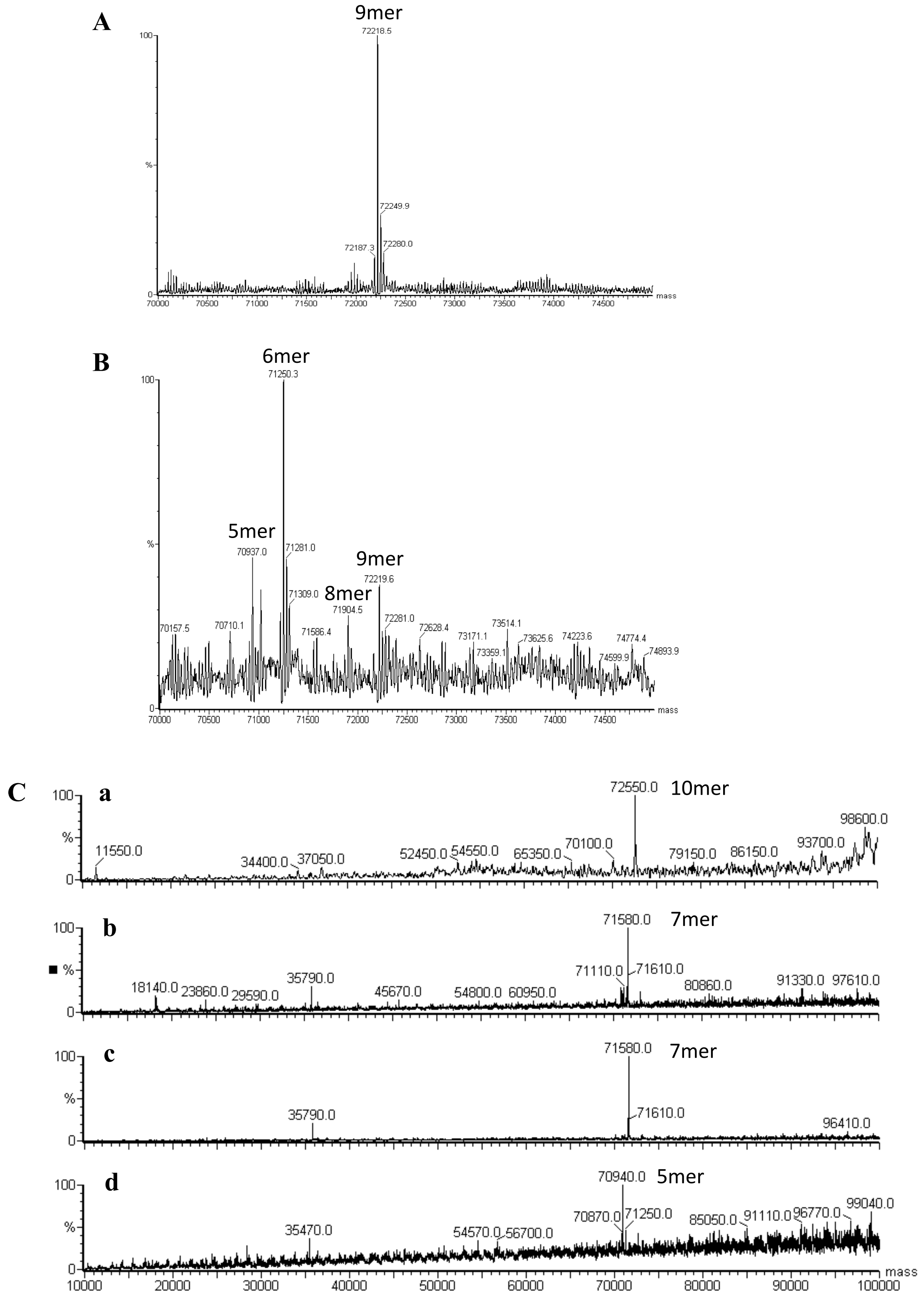

Figure S3. **(A)** Intact mass spectrometry of XPD-9mer covalent complex after overnight incubation of XPD with the DNA, in the absence of AMPPNP/$MgCl_2$. **(B)** The XPD-9mer covalent complex was incubated after anion exchange (AE) purification with 3 mM AMPPNP and 5 mM $MgCl_2$ overnight. The species corresponding to the complexes with the calculated lengths of DNA based on the molecular mass are indicated. **(C)** Intact mass spectrometry of: **(a)** XPD-10mer intact covalent complex; **(b)** XPD-10mer complex incubated with 10 mM AMPPNP & 10 mM $MgCl_2$ overnight; **(c)** apo XPD + 10mer DNA incubated as in **(b)**; **(d)** apo XPD + 10mer incubated with 10 mM $MgCl_2$ in the absence of AMPPNP overnight.

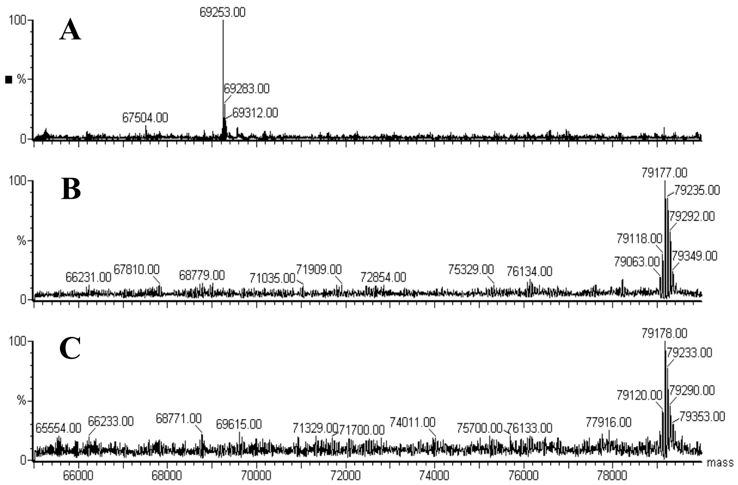

Figure S4. Intact mass spectrometry of apo XPD **(A)** and XPD – hairpin DNA covalent complex after overnight incubation of XPD with the DNA **(B)**. The bottom panel **(C)** represents a sample in which XPD was incubated with the DNA in the presence of 3 mM AMPPNP and 5 mM $MgCl_2$ overnight. The mass is similar to the one in panel **B**, so the hairpin DNA remains intact.

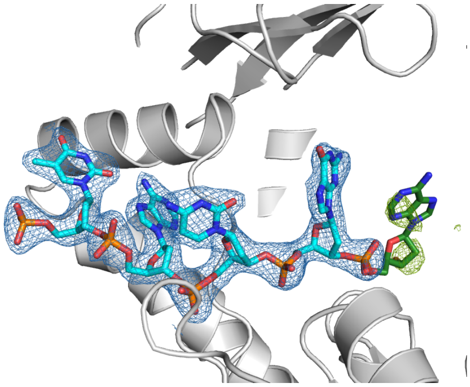

Figure S5. 2Fo-Fc map contoured at 1 σ (blue) of the modeled DNA and Fo-Fc density contoured at 3 σ (green) of the 5th nucleotide that could not be properly modeled.

Scheme S1. Skeletal formula of $BM(PEG)_3$

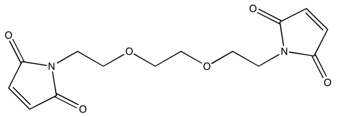

1, 11-bis (maleimido) triethylene glycol
$BM(PEG)_3$

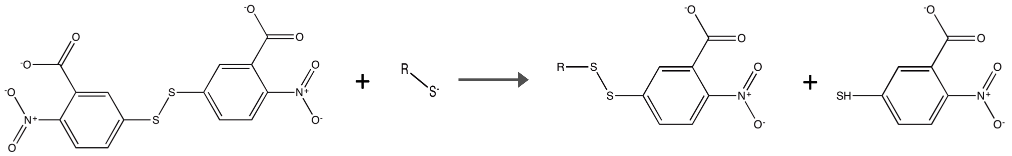

Scheme S2. Schematic representation of the reaction between the DTNB reagent and sulfhydryl.

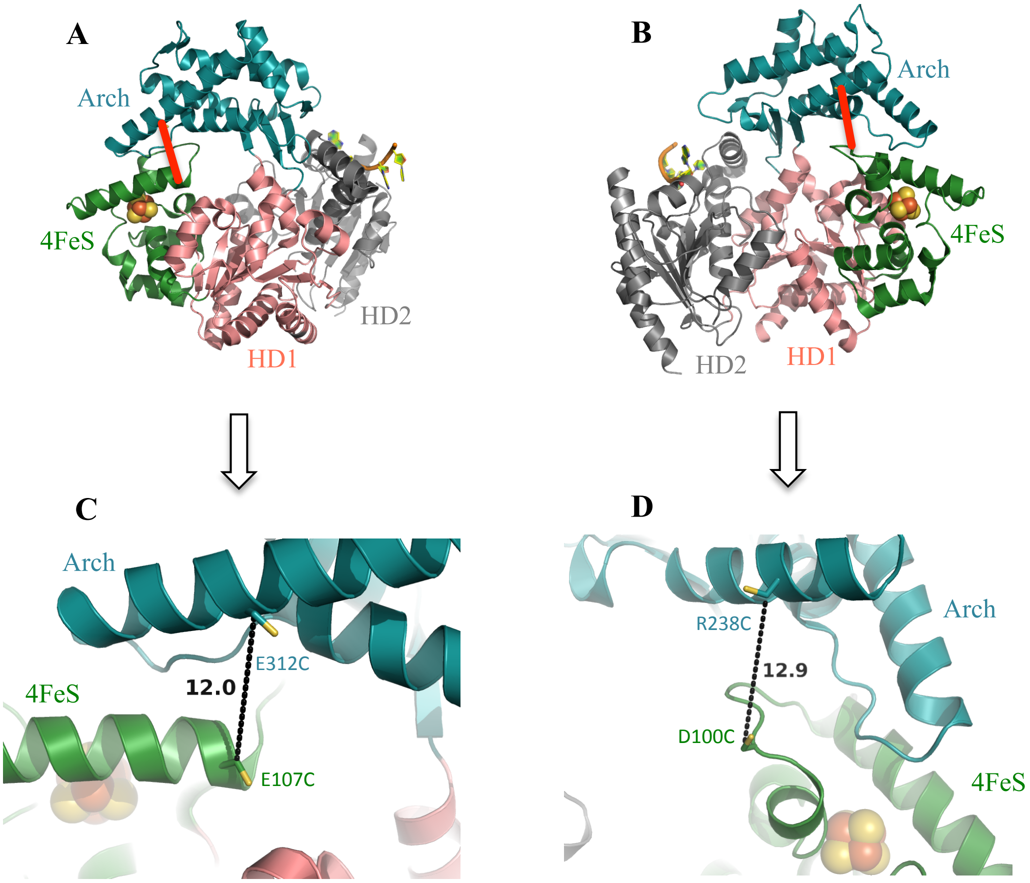

Figure S6. The cysteine mutants pairs used for the intra-molecular crosslinking of Arch domain to 4FeS domain (represented as red bars in panels **A** and **B**) and the corresponding distances between Cβ: **(A, C)** E107C (4FeS domain) – E312C (Arch domain) (12 Å) and **(B, D)** D100C (4FeS domain) – R238C (Arch domain) (12.9 Å).

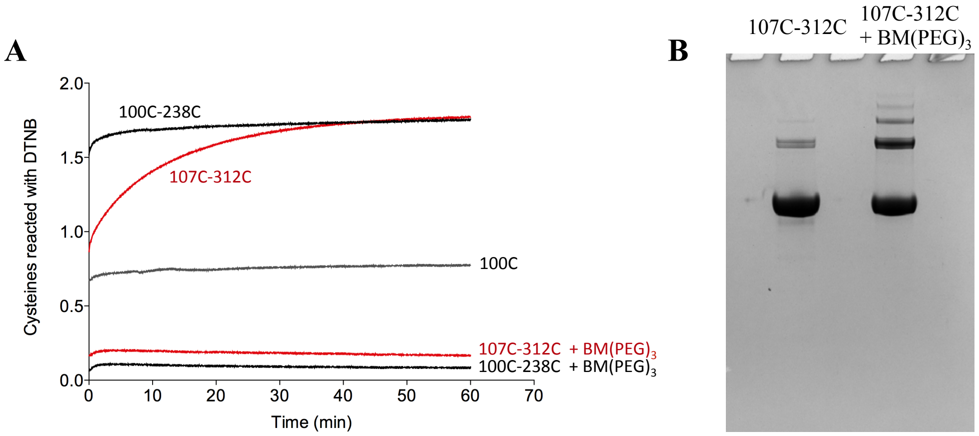

Figure S7. (A) Reaction of XPD (10 μM) with DTNB (40 μM). The time course of the absorption at 412 nm was recorded and scaled as equivalents of reacted cysteines. **(B)** SDS-PAGE of XPD 107C-312C ± $BM(PEG)_3$. All samples were subjected to the same experimental conditions.

Table S1. Number of free cysteines quantified from DTNB assay. The values for the cysteine mutants include the subtraction of the "no cysteine" value. *The values represent the results of two different crosslinking protocols (see Material and Methods).

XPD	Cysteine-TNB
No cysteine	0.13
100C	0.8
100C-238C	1.75
100C-238C + $BM(PEG)_3$	0.3/0.08*
107C-312C	1.77
107C-312C + $BM(PEG)_3$	0.17

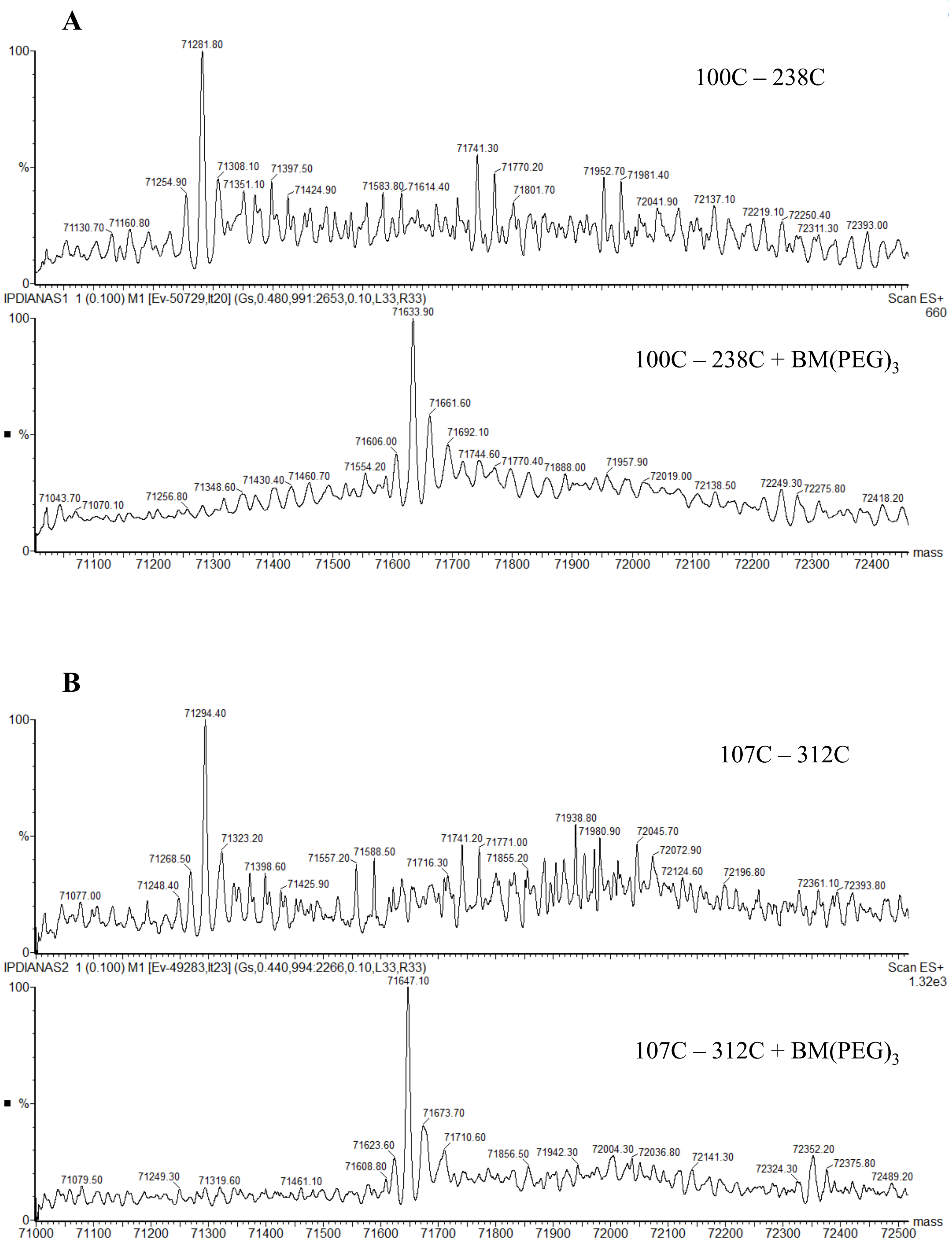

Figure S8. Mass spectrometry of intra-molecularly crosslinked XPD 100C – 238C **(A)** and 107C – 312C **(B)** double mutants. The non-crosslinked double mutants are shown as control. The theoretical additional mass for one molecule of BM(PEG)$_3$ crosslinker is 352.34 Da.

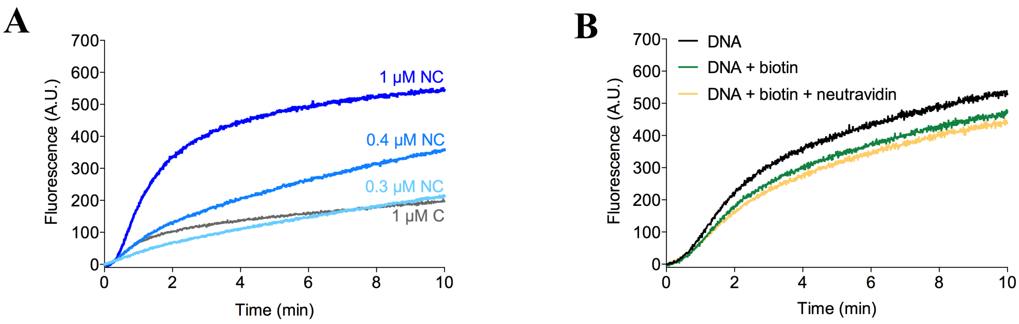

Figure S9. (A) The helicase activity of the non-crosslinked (NC) XPD 107C-312C as a function of concentration and the activity of 1 μM crosslinked (C) XPD 107C-312C. **(B)** Helicase unwinding of 50 nM fork DNA by 0.5 μM XPD 100C-238C in the absence and presence of biotin ± neutravidin modification.
